# Improvement of Phenolic Bioaccessibility and Gut Microbiota Modulation Potential of Black Rice by Extrusion Combined with Solid-State Fermentation

**DOI:** 10.3390/foods15010032

**Published:** 2025-12-22

**Authors:** Chunyan Bo, Ersheng Gong, Liqiang Zou, Yejun Zhong, Jinshen Chu, Jianyong Wu, Fangqing He, Zicong Zeng

**Affiliations:** 1 Jiujiang City Key Laboratory of Cell Therapy, Jiujiang NO.1 People’s Hospital, Jiujiang 332000, China; bochunyan0126@126.com (C.B.); chujinshen@126.com (J.C.); 2 School of Food Science and Engineering, Jiangxi Agricultural University, Nanchang 330045, China; 3 State Key Laboratory of Food Science and Resources, Nanchang University, Nanchang 330047, China; zouliqiang2010@163.com (L.Z.); jianyongwu@ncu.edu.cn (J.W.); 417900210051@email.ncu.edu.cn (F.H.); 4 Key Laboratory of Prevention and Treatment of Cardiovascular and Cerebrovascular Diseases (Ministry of Education), School of Public Health and Health Management, Gannan Medical University, Ganzhou 341000, China; gongersheng@163.com

**Keywords:** black rice, extrusion, solid-state fermentation, phenolic bioaccessibility, gut microbiota

## Abstract

Black rice has gained increasing attention due to its abundant phenolic compounds and gut microbiota modulation potential, but its health benefits are highly dependent on processing methods. In the present study, the effects of extrusion, traditional cooking, and their combinations with solid-state fermentation (SSF) on phenolic bioaccessibility and the gut microbiota modulation potential of black rice were compared. Results indicated that extrusion combined with SSF (E-SSF) was the most prominent in improving the bioaccessibility of phenolics, flavonoids, and antioxidant activities of black rice during in vitro gastrointestinal digestion. In addition, black rice after SSF induced significantly lower gas production and higher pH during in vitro fecal fermentation. Particularly, black rice after E-SSF showed great advantages in the yield of propionic acid, butyric acid, and total short-chain fatty acids. Consequently, black rice after SSF increased alpha diversity and Bacteroidetes abundance but decreased Firmicute abundance of gut microbiota, while black rice after E-SSF induced the highest alpha diversity and Bacteroide abundance. These results suggested that SSF was beneficial to improve the gut microbiota modulation potential of black rice, and E-SSF was the most preferred. In conclusion, E-SSF was the most suitable to improve the phenolic bioaccessibility and gut microbiota modulation potential of black rice.

## 1. Introduction

Black rice (*Oryza sativa* L.) is a typical whole grain with a long history of cultivation and consumption in Asian countries, and has always been used as a medicinal herb and food. [1]. It has been reported to be abundant in bioactive compounds (e.g., phenolic compounds) which have significant health benefits in the prevention of inflammation, type II diabetes, cardiovascular disease, cancers, etc. [1,2]. Thus, black rice has gained increasing public attention due to its abundant nutrients and therapeutic nature. In addition, plenty of studies have also proved that black rice is an abundant source of prebiotics (e.g., dietary fiber, polyphenols) and showed great potential in the modulation of gut microbiota and thereby influence the overall human health [3].

Currently, black rice is frequently exposed to a series of processes, including conventional hydrothermal cooking, air heating, steaming, extrusion, emerging superheated steam, infrared irradiation, and microwave treatment to improve its sensory, nutritional, or storage qualities [4]. Extrusion is outstanding among the above processing methods due to its continuous production, high productivity, versatility, and low cost, and thereby is widely used in the food industry [5]. Nevertheless, the effects of extrusion on the nutritional properties were not so desirable, and it may negatively damage the phytochemicals (e.g., phenolic compounds) of black rice [6]. Furthermore, only bioaccessible phenolics released from the food matrix and solubilized in the digestive fluid are able to exert their bioactivities. Therefore, new methods or combined treatments based on the merits of various processing techniques are highly encouraged to improve the phenolic bioaccessibility of extruded black rice.

Solid-state fermentation (SSF) is a bioconversion process in which the microorganisms grow on low-moisture solid-state substrates with the absence (or near absence) of free water [7]. Numerous studies have indicated that SSF is a favorable strategy to improve the cooking, eating, and phenolic compounds of black rice [4,8]. In addition, filamentous fungi (e.g., *Aspergillus oryzae*) are the most recommended for SSF among different microorganisms since SSF is approximate to their natural habitat [4]. In addition, it was reported that extrusion pretreatment could effectively improve the fermentation efficiency of whole grains, and then fermentation was able to break the covalent bonds of dietary fiber and change its structure [9]. As a result, the colonic fermentability of dietary fiber might be greatly improved and thereby influence the gut microbiota modulation potential of black rice [9,10].

It is worth noting that the nutritional properties of black rice are highly associated with gut microbiota since the intake of black rice could increase healthy gut microbiota by adjusting their richness, diversity, and short-chain fatty acid (SCFA) production [11]. Previous studies have demonstrated that the composition of the gut microbiota and their metabolites have profound effects on the energy homeostasis, lipid and glucose metabolism, inflammation, and cancerization of host, which are beneficial for maintaining the overall health of host [3]. Therefore, we hypothesize that extrusion combined with SSF might be more suitable to improving the phenolic bioaccessibility and gut microbiota modulation potential of black rice, which is significant for improving the nutritional properties of black rice.

In this study, extrusion combined with SSF was applied to improve the phenolic bioaccessibility and gut microbiota modulation potential of black rice, and the effects were compared with those of extrusion, traditional cooking, and traditional cooking combined with SSF. Specifically, the bioaccessibility of phenolics, flavonoids, antioxidant activities, and the bioaccessible phenolic acids of different black rice samples during in vitro simulated digestion were investigated. Afterwards, the gas production, pH, SCFAs, and microbial communities incubated with different black rice samples during in vitro simulated large bowel fermentation were also analyzed. The present study will provide a favorable strategy to improve the functional properties of black rice and then promote its consumption and application in the food industry.

## 2. Materials and Methods

### 2.1. Materials and Chemicals

Black rice (Heibao) was purchased from Guangxi Bama Qianbainian Health Food Co. Ltd. (Bama Yao Autonomous County, China) and stored at −20 °C until used. It was grown at 24 N latitude (Guangxi Bama) from March to July in 2022. The bacteria powder for fermentation (*Aspergillus oryzae*, 10^7^–10^8^ spores/g) was from Suzhou Ten Acres Small Yard Ecological Agriculture Co., Ltd. (Suzhou, China). Substances used in the in vitro digestion, including pepsin (porcine gastric mucosa origin), lipase (porcine pancreas origin), and pancreatin (porcine pancreas origin), were purchased from Sigma-Aldrich (Shanghai, China), and bile salt was from Shanghai Macklin Biochemical Co., Ltd. (Shanghai, China). In addition, the standards of phenolic acids, antioxidant reagents, resazurin, hemin, and vitamin K1 all originated from Aladdin Industrial Inc. (Shanghai, China). Other reagents used were from Xilong Chemical Co., Ltd. (Shantou, China), and their purities were either analytical or HPLC grade.

### 2.2. Black Rice Preparation and Subsequent Solid-State Fermentation

#### 2.2.1. Preparation of Black Rice Samples

The extruded black rice was prepared based on our previous study with minor modifications [12]. In detail, raw black rice was first milled and passed through a 40-mesh screen in a universal grinder (STWS-40, Shengtian Machine Manufacturing Co., Ltd., Jiangyin, China). Afterwards, the moisture content of black rice powder was adjusted to 30% and then processed by a twin-screw extruder (FMHE36-24, Hunan Fumake Food Engineering Technology Co., Ltd., Changsha, China). The temperatures of different zones were set as 70–100–170–170–170 °C, the feed rate was maintained at 8.0 kg/g, the main screw speed was set as 130 rpm, and the speed of the rotary cutter was 1800 rpm. In addition, the traditional cooked black rice was also prepared with an electric cooker (ZCDQ055, Chunmi Technology Co., Ltd., Shanghai, China). The black rice was cooked for 40 min with the ratio of black rice to water being 1:2 (*w*/*w*). Then, both the extruded black rice (EBR) and cooked black rice (CBR) were dried at 45 °C for 24 h with a hot-air drying oven (GZX-9420MBE, Shanghai Boxun Industry Co., Ltd., Shanghai, China), and then the dried black rice samples were stored at −20 °C until used.

#### 2.2.2. Solid-State Fermentation (SSF) of Black Rice Samples

The SSF of black rice was performed according to our previous study [4]. A suitable amount (10 g) of different black rice samples was put into petri dishes with lids, and then exposed to UV lights in a super-clean workbench for 30 min for sterilization purposes. Subsequently, the moisture of sterilized EBR and CBR was adjusted to 40% with the addition of sterile water, and then 20 mg of bacteria powder of *Aspergillus oryzae* (10^7^–10^8^ spores/g) was inoculated into the above black rice samples, fermented at 35 °C and 90% humidity for 28 h, followed by fermentation at 55 °C for 5 h. Finally, the fermented extruded black rice (FEBR) and fermented cooked black rice (FCBR) were dried at 45 °C for 24 h in a hot-air drying oven, and then all the black rice samples (EBR, CBR, FEBR, and FCBR) were milled, passed through a 40-mesh screen, and stored at −20 °C until used.

### 2.3. In Vitro Simulated Oral-Gastric-Intestinal Phase Digestion

#### 2.3.1. Preparation of Simulated Digestive Fluid

Simulated digestive fluids used in the present study, including simulated salivary fluid, simulated gastric fluid, and simulated intestinal fluid, were prepared according to the methods in our previous study [13]. Afterwards, the above simulated digestive fluids were stored at −20 °C and mixed with a suitable amount of 0.3 mol/L CaCl_2_(H_2_O)_2_ and enzymes just before use.

#### 2.3.2. In Vitro Simulated Digestion

The in vitro simulated digestion was performed by using a three-stage digestive model (i.e., oral-gastric-intestinal phase) based on a previous study with slight modifications [13].

Oral phase: 3.0 g of black rice samples prepared in Section 2.2.2 was mixed evenly with 10 mL distilled water, 8 mL simulated salivary fluid, and 50 μL 0.3 mol/L CaCl_2_(H_2_O)_2_, and the pH of the mixture was adjusted to 7.0. Subsequently, 50 μL 3000 U/mL α-amylase was added to the mixture to initiate the oral digestion, and then incubated at 37 °C for 10 min in a shaking water bath in the dark. In addition, a blank test was also performed by replacing the black rice samples with an equal amount of distilled water.

Gastric phase: the oral digesta was immediately mixed with 16 mL simulated gastric fluid and 10 μL 0.3 mol/L CaCl_2_(H_2_O)_2_, and the pH of the mixture was adjusted to 3.0 with 1 mol/L HCl. Afterwards, 1.6 mL 20 mg/mL pepsin solution, 0.3 mL 80 mg/mL lipase solution, and a suitable amount of distilled water were added to start the digestion, and then incubated at 37 °C for 2 h in a shaking water bath in the dark.

Intestinal phase: before intestinal digestion, a segment of dialysis bag (molecular mass cut off: 8000–14,000 Da) was rinsed with 0.9% NaCl and then filled with 2.5 mL 0.9% NaCl and 2.5 mL 0.2 mol/L NaHCO_3_. Thereafter, the dialysis bag was tied tightly at both ends and immersed in the residual gastric digesta immediately after gastric digestion. The mixture was still incubated at 37 °C for another 45 min till the pH increased to c.a. 6.5 to simulate the transitory stage from the gastric phase to the intestinal phase. After that, 14 mL of simulated intestinal fluid and 70 μL of 0.3 mol/L CaCl_2_(H_2_O)_2_ were added, and the pH of the digesta was adjusted to 7.0 with 1 mol/L NaOH. Subsequently, 5 mL 75 mg/mL bile salt solution, 8 mL 24.5 mg/mL pancreatin solution, and a suitable amount of distilled water were added to the mixture, followed by incubation at 37 °C for 2 h in a shaking water bath in the dark to simulate intestinal digestion.

When the gastric or intestinal digestion finished, 5 mL digesta was withdrawn and immediately mixed thoroughly with an equal amount of methanol to inactive enzymes, followed by centrifugation, and the supernatants together with dialysate were collected. The pH of all collected samples (i.e., gastric digesta, intestinal digesta, and dialysate in the bag) was adjusted to 7.0 and stored at −20 °C until further analysis. In addition, the residual digesta outside the bag after intestinal digestion was filled in another dialysis bag (molecular mass cut off: 1000 Da) and dialyzed in ultrapure water for 72 h, with the water changed every 6 h to further remove the small molecular substances. Then, the non-dialyzed fraction in the bag was lyophilized, crushed, and stored at −20 °C till the use of in vitro fecal fermentation.

#### 2.3.3. In Vitro Digestibility of Phenolic Compounds

During in vitro simulated digestion, the phenolic compounds in black rice showed different release behaviors in gastric and intestinal digestion, and then the phenolic compounds became bioaccessible in the dialysate. Therefore, the phenolic contents, flavonoid contents, and antioxidant activities of gastric digesta, intestinal digesta, and dialysate (collected in Section 2.3.2) were firstly determined by the Folin–Ciocalteu method, the AlCl_3_ colorimetric method, and FRAP and DPPH assays, respectively [13]. Afterwards, the phenolic compounds recovered in the dialysate represented the bioaccessible fraction, and then their bioaccessibilities were calculated by the ratio of bioaccessible fraction to the amount in raw samples before digestion. Specifically, the bioaccessible phenolic acids (representative phenolic compounds in whole grains) were also investigated by the HPLC method according to our previous study [13]. In detail, chromatographic separation was performed with a reverse-phase C18 column (250 × 4.6 mm; 5 µm; SunFire, Waters, Milford, MA, USA) under an Agilent 1260 HPLC system equipped with a variable wavelength detector. The 1.3% glacial acetic acid in water (solvent A) and 100% acetonitrile (solvent B) were used as mobile phase, and the gradient eluted program was as follows: 0–2 min, 10% B; 2–20 min, 10–13% B; 20–35 min, 13–20% B; 35–45 min, 20–35% B; 45–80 min, 35–45% B; 80–93 min, 45–100% B; 93–98 min, 100–8% B; 98–105 min, 8% B. Additionally, injection volume, flow rate, and detector wavelength were set as 20 μL, 0.8 mL/min, and 280 nm, respectively. Finally, the individual phenolic acids were determined based on the retention time and standard curve of authentic standards.

### 2.4. In Vitro Simulated Large Bowel Fermentation and Metabolite Analyses

#### 2.4.1. In Vitro Fecal Fermentation

The lyophilized residues obtained in Section 2.3.2 were used for in vitro large bowel fermentation by using human fecal microbiota described in a previous study [14]. The human fecal samples used were obtained from four adult male healthy donors (21–25 years old) with their written informed consent. Fresh fecal samples were collected from healthy participants who had no usage records of antibiotics for the past 6 months and followed their routine diet. Then, the fecal samples were immediately transferred into an anaerobic workstation (Bactron300-2, Shellab Bactron Company, Cornelius, NC, USA), followed by dispersion with three volumes of basal culture medium by vortex mixing. Afterwards, the slurries were filtered through four layers of cheesecloth to remove visible particles. Thereafter, 50 mg digested black rice samples were mixed with 0.2 mL fecal slurry in a serum bottle containing 4.8 mL basal culture medium. All the above steps were performed in anaerobic conditions with 80% N_2_, 10% CO_2_, and 10% H_2_, then the sealed serum bottles were incubated at 37 °C and 70% humidity. The samples were collected at 0, 3, 6, 12, and 24 h, respectively, and then investigated by their gas production, pH, and short-chain fatty acids (SCFAs). In addition, the blank control sample was also performed by replacing the digested black rice samples with sterile water. Based on the description of human samples in the “Measures for Ethical Review of Life Science and Medical Research Involving Humans” promulgated by the National Health Commission of the People’s Republic of China: The utilization of human fecal samples in this study complies with the following ethical criteria: (1) the experimental procedures involving these samples pose no direct or indirect harm to human health; (2) the collection and analysis of samples do not entail the disclosure of sensitive personal information, privacy, or commercial interests; (3) the research objectives and methodologies are fully aligned with the principles of informed consent. Furthermore, this study does not involve the use of human reproductive cells, embryos, reproductive cloning, chimerism, or heritable genetic modifications. Confirmed with the Ethics Committee of Jiujiang NO.1 People’s Hospital, this study is exempt from ethical review.

#### 2.4.2. Determination of Gas Production, pH, and SCFAs

The gas production of cultured samples at different time points was measured by a syringe. Afterwards, the cultured samples were centrifuged, and the supernatants were collected, then the pH was determined by a micro pH meter (13-620-290, Thermo Fisher Scientific, USA). In addition, 400 μL supernatant was mixed evenly with 45 μL ultrapure water, 80 μL 1 mol/L hydrochloric acid, and 65 μL 10 mmol/L 2- ethylbutanoic acid by vortexing, followed by centrifugation at 4 °C, 12,500 rpm for 5 min. Subsequently, the supernatants were collected and filtered through a 0.22 μm filter membrane, and then the filtrate was analyzed by a gas chromatograph (7890 B, Agilent Technology Co., Ltd., Santa Clara, CA, USA) according to a previous study for qualitative and quantitative analysis of SCFAs [15].

#### 2.4.3. The 16S rRNA Amplicon Sequencing of Gut Microbiota

DNA was extracted from the precipitates isolated from the fermented samples by using OMEGA Soil DNA Kit (M5635-02) (Omega Bio-Tek, Norcross, GA, USA), as described in a previous study [16]. The concentration and purity of extracted DNA were confirmed by using a NanoDrop NC2000 spectrophotometer (Thermo Fisher Scientific, Waltham, MA, USA) and agarose gel electrophoresis, respectively. Subsequently, the V3-V4 region of the 16S rRNA gene was amplified by PCR with the forward primers of 338F (5′-barcode+ACTCCTACGGGAGGCAGCA-3′) and reverse primer 806R (5′-GGACTACCAGGGTATCTAAT-3′). Then, the amplicons were extracted by 2% agarose gel electrophoresis and purified by the Axygen Gel DNA recovery kit (Axygen Biotechnology (Hangzhou) Co., Ltd., Hangzhou, China), quantified by the Quant-iT PicoGreen dsDNA Assay Kit (Invitrogen, Carlsbad, CA, USA). Thereafter, all PCR amplicons were combined in equal amounts and pair-end sequenced (2 × 250 bp) on the Illumina MiSeq platform with the MiSeq Reagent Kit v3 at Shanghai Personal Biotechnology (Shanghai, China).

#### 2.4.4. Microbiome Bioinformatics

The microbiome bioinformatics were analyzed by Quantitative Insights Into Microbial Ecology (QIIME2), according to a previous study [17]. Firstly, raw sequence data were demultiplexed by using the demux plugin and then primer clipped by using the cutadapt plugin. Then, the sequences were quality filtered, denoised, merged, and chimera removed by using the DADA2 plugin, which resulted in a table of amplicon sequence variants (ASVs) and their abundances. Subsequently, non-singleton ASVs were aligned with mafft and used to create a phylogeny with fasttree2. Alpha diversity metrics (e.g., Shannon index, observed features index, and Pielou’s evenness index) and beta diversity metrics were performed using the diversity plugin. A taxonomy was assigned to ASVs using the Klearn naive Bayes taxonomy classifier in the Feature Classifier plugin, while the Greengenes database (v.13.8) was used as the reference database.

### 2.5. Statistical Analysis

All the data were acquired from at least three repeated determinations, and the results are presented as mean ± standard deviation (SD). Afterwards, the results were statistically analyzed by using SPSS software (version 26.0, SPSS Inc., Chicago, IL, USA) and R 4.1.3 using one-way analysis of variance (ANOVA) and Tukey’s post hoc test, and the significant level was set as *p* < 0.05. The LEfSe analysis was performed at the ASVs level with linear discriminant analysis (LDA) score larger than 4.0.

## 3. Results

### 3.1. Phenolic Bioaccessibility of Black Rice During In Vitro Gastrointestinal Digestion

#### 3.1.1. Variation of Phenolics, Flavonoids, and Antioxidant Activities

Phenolics and flavonoids are supposed to be the major bioactive compounds in black rice, and their contents and compositions are significantly influenced by the processing methods and subsequent digestion [13]. Generally, the phenolic compounds in foods will experience a series of enzymatic reactions during digestion, the free and conjugated phenolics in the food matrix will be released to the digestive fluids, and the bound phenolics will mainly survive from the gastrointestinal digestion, resulting in a variation in phenolics, flavonoids, and antioxidant activities [18,19].

**Figure 1:**
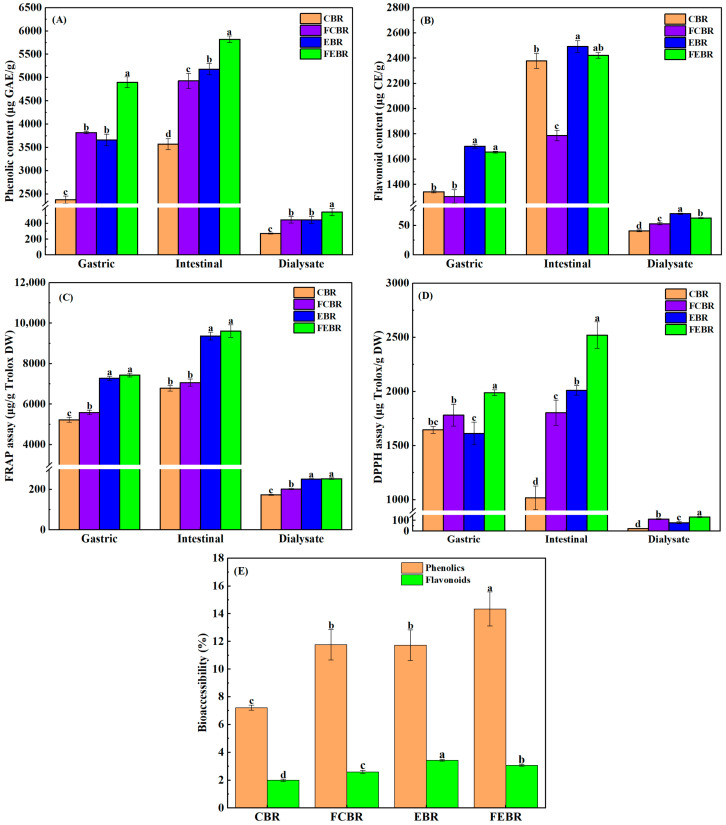
The phenolics, flavonoids, and antioxidant activities of different black rice samples during in vitro gastrointestinal digestion. (**A**) Phenolic content; (**B**) flavonoid content; (**C**,**D**) antioxidant activities determined by FRAP and DPPH assays, respectively; (**E**) bioaccessibility of phenolics and flavonoids. Values labeled with different letters in the same digestion phase differ significantly (*p* < 0.05).

The phenolics, flavonoids, and antioxidant activities of different black rice samples during in vitro digestion are shown in Figure 1A–D. After gastric digestion, the phenolic content in fermented extruded black rice (FEBR) was 4899.4 μg GAE/g DW, but it was significantly decreased to 3820.3 μg GAE/g DW in fermented cooked black rice (FCBR), and even further to 3661.3 and 2378.5 μg GAE/g DW, respectively, in extruded black rice (EBR) and cooked black rice (CBR) (Figure 1A). It could be found that the phenolic content in EBR was significantly higher than that in CBR, which was mainly because the disruption of black rice cell wall during the harsh conditions (e.g., high temperature, high pressure, and high shearing force) of extrusion facilitated the gastric digestion [20]. In addition, fermented black rice samples also showed significantly higher phenolic content than that of unfermented ones, which was probably due to the release of phenolics during solid-state fermentation [21].

Furthermore, the flavonoid contents in different black rice samples after gastric digestion are given in Figure 1B. It could be found that the flavonoid content was the highest in EBR (1703.4 μg CE/g DW), followed by FEBR (1655.9 μg CE/g DW), and then significantly decreased to 1304.7 and 1341.2 μg CE/g DW, respectively, in CBR and FCBR. The above results are slightly different from those of phenolic contents (Figure 1A), which is probably due to the sensitivity of flavonoids to pH and digestive enzymes [22,23]. Afterwards, the antioxidant activities of different black rice samples after gastric digestion were determined by FRAP and DPPH assay (Figure 1C,D). The results indicated that FEBR had the highest antioxidant activities among all black rice samples in different assays, which was mainly due to its higher contents of phenolics and flavonoids as aforesaid.

Moreover, the variation in phenolics, flavonoids, and antioxidant activities of different black rice samples after in vitro intestinal digestion was also investigated, and the results are presented in Figure 1A–D. It could be seen that the phenolics, flavonoids, and antioxidant activities in all samples were significantly increased after intestinal digestion when compared with their corresponding contents after in vitro gastric digestion. It has been reported that the phenolic compounds might be continuously released from the food matrix with the effects of pH, bile salts, digestive enzymes, and the extension of digestion time. It was probably due to the degradation of macromolecules and destruction of interactions between phenolic compounds and the food matrix during intestinal digestion [18]. In addition, it was worth noting that EBR and FEBR showed significantly higher phenolics, flavonoids, and antioxidant activities than CBR and FCBR after in vitro intestinal digestion, and FEBR was the most prominent, indicating that extrusion combined with solid-state fermentation was the most valuable to improve the phenolic digestibility.

#### 3.1.2. Bioaccessibility of Phenolics, Flavonoids, and Phenolic Acids

During gastrointestinal digestion, phenolic compounds are released from the food matrix and solubilized in digestive fluids, and subsequently become bioaccessible in dialysate [13]. The bioaccessible phenolics, flavonoids, and antioxidant activities in different black rice samples are presented in Figure 1A–D. It was found that FEBR showed significantly higher bioaccessible phenolics (544.5 μg GAE/g DW) than other samples, while its bioaccessible flavonoids were significantly lower than those in EBR, which was consistent with their contents after gastric and intestinal digestion. Consequently, the dialysate of FEBR showed the highest antioxidant activity with the FRAP and DPPH antioxidant activities of 252.9 and 131.1 μg Trolox/g DW, respectively. Afterwards, the bioaccessibility of phenolics and flavonoids was also calculated, and the results are shown in Figure 1E. The results demonstrated that the bioaccessibilities of phenolics were 7.21%, 11.76%, 11.73%, and 14.35%, respectively, in CBR, FCBR, EBR, and FEBR. In addition, the bioaccessibilities of flavonoids were 2.00%, 2.60%, 3.43%, and 3.07%, respectively, in CBR, FCBR, EBR, and FEBR. The above results clearly indicated that the bioaccessibilities of phenolics and flavonoids were relatively low in black rice, which was consistent with the previously reported bioavailability (5–10%) of phenolic compounds in the human digestive tract [19]. Specifically, the bioaccessibilities of phenolics in FCBR, EBR, and FEBR were more than 10%, and the FEBR showed the highest bioaccessibility of phenolics. Therefore, it could be concluded that extrusion combined with solid-state fermentation (E-SSF) was the most valuable in improving the phenolic bioaccessibility.

Furthermore, phenolic acids have been reported to be the most abundant phenolic compounds in whole grains, and they are the primary metabolites of other polyphenol metabolism prior to absorption or degradation during intestinal digestion [23]. Therefore, the bioaccessible phenolic acids of different black rice samples during in vitro intestinal digestion were also investigated, and the results are shown in Table 1. The results demonstrated that the phenolic acid profile of different black rice samples after intestinal digestion mainly consisted of *p*-hydroxybenzoic acid, vanillic acid, and caffeic acid, and *p*-hydroxybenzoic acid was the most abundant in all black rice samples. In detail, it was 662.6, 670.8, 658.2, and 664.4 μg/g DW, respectively, in CBR, FCBR, EBR, and FEBR, which consisted of 69.4%, 67.9%, 66.2%, and 66.2%, respectively, of their corresponding total phenolic acids. The above results were similar to a previous study in which some colored plant foods had higher content of *p*-hydroxybenzoic acid [24], and more *p*-hydroxybenzoic acid might be released with the effects of bile salts and digestive enzymes during intestinal digestion. In addition, it was found that the contents of vanillic acid, *trans*-sinapic acid, and total phenolic acids in FEBR were significantly higher than those of other black rice samples after in vitro intestinal digestion. Moreover, the phenolic acid profile in dialysate of different black rice samples was highly related to their corresponding contents after intestinal digestion, and *p*-hydroxybenzoic acid showed the highest bioaccessibility among all phenolic acids. In addition, the content of total phenolic acids in dialysate was 43.1, 44.8, 44.8, and 49.2 μg/g DW, respectively, in CBR, FCBR, EBR, and FEBR, in which FEBR showed significantly higher content of total phenolic acids than that of other black rice samples. Therefore, it could be concluded that E-SSF was the most beneficial to improve the bioaccessibility of phenolic acids.

### 3.2. Gut Microbiota Modulation Potential of Black Rice During In Vitro Fecal Fermentation

The digestive chyme of black rice consisted of abundant carbohydrates, proteins, and phytochemicals. These major components could be released and partially absorbed during gastrointestinal digestion, followed by the fermentation of gut microbiota in the cecum and colon. During fecal fermentation, a crucial metabolite (SCFAs) will be generated and thereby beneficial to intestinal health and further affect human health [3].

#### 3.2.1. Gas Production and pH Variation

**Figure 2:**
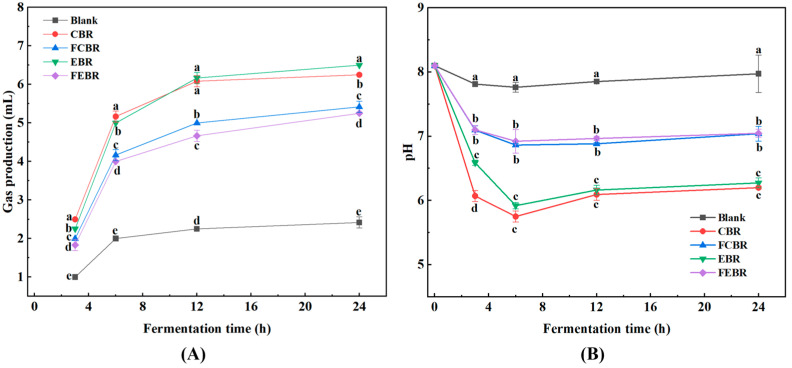
The gas production and pH of different black rice groups during in vitro large bowel fermentation. (**A**) Gas production; (**B**) pH. Values labeled with different letters at the same fermentation time differ significantly (*p* < 0.05).

Gas production is a commonly used indicator that demonstrates the fermentability of carbohydrates. The gas accumulation in different black rice groups during in vitro fecal fermentation is depicted in Figure 2A. It can be seen that the gas accumulation in all black rice groups, except the blank control group, increased rapidly in the first 6 h of fermentation, indicating that black rice substrates were well utilized by the gut microbiota. Afterwards, the gas production turned to decline from 6–12 h, and then tended to nearly stop after 24 h of fermentation, indicating the utilization of the nutrients in the black rice substrates was almost complete. It was worth noting that the gas accumulation of fermented black rice groups (FCBR and FEBR) was significantly lower than that of unfermented black rice groups (CBR and EBR). It has been reported that the intake of whole grains rich in fermentable oligosaccharides could increase the risk of flatulence [25]. Thus, the decrease in gas accumulation in FCBR and FEBR was beneficial to the prevention of flatulence, and it was probably because the conversion of black rice substrates from macromolecules unutilized in the gastrointestinal tract to absorbable small molecules, resulting in the decrease in fermentable compounds.

The pH of the fermentation substrate provides a basic environment for the growth of microorganisms, and a low pH environment in the gut was reported to be beneficial to intestinal health [26]. As shown in Figure 2B, the variation in pH values with increasing fermentation time was consistent with the changes in gas production. In detail, the pH of all black rice groups was first decreased from 0–6 h, and then became steady from 6–24 h. The initial decrease in pH was probably due to the production of SCFAs and other microbial metabolites during fermentation, while the subsequent increase in pH might result from the generation of methane or ammonia in later fermentation [15]. In addition, it could also be found that the pH value of the blank control group after 24 h of fermentation was very close to the initial value, while the pH values of all black rice groups were obviously lower than their corresponding initial values after 24 h of fermentation. Particularly, it was found that the pH values of unfermented black rice groups (CBR and EBR) were significantly lower than those of fermented black rice groups (FCBR and FEBR). The possible reason for the above results was that the carbohydrates in fermented black rice were severely degraded into small-molecule monosaccharides and then dialyzed off in the in vitro gastrointestinal digestion. Consequently, the remaining few oligosaccharides and polysaccharides in fermented black rice samples induced less production of acidic compounds and then increased the pH values.

#### 3.2.2. SCFA Production

**Figure 3:**
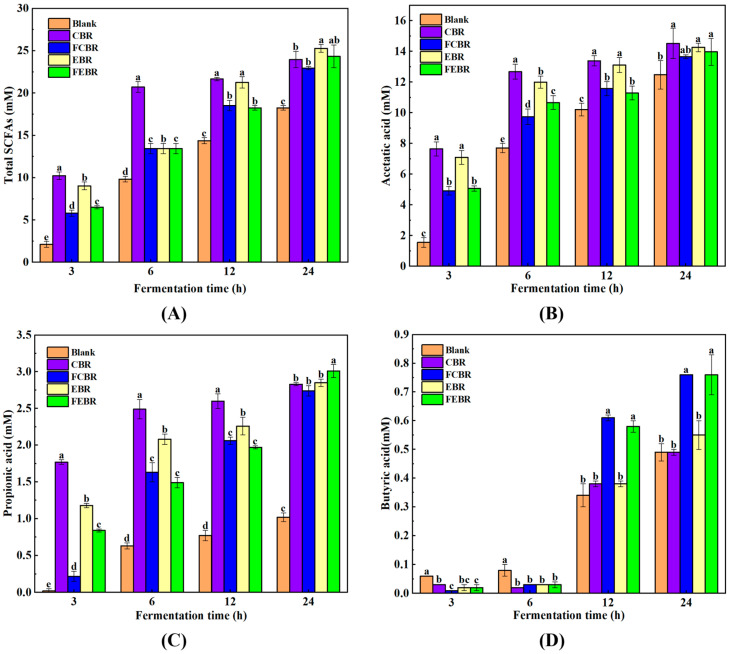
The SCFAs of different black rice groups during in vitro fecal fermentation. (**A**) Total SCFAs; (**B**) acetic acid; (**C**) propionic acid; (**D**) butyric acid. Values labeled with different letters at the same fermentation time differ significantly (*p* < 0.05).

Generally, SCFAs are generated by the anaerobic microorganisms in the colon and play important roles in the intestinal homeostasis and human health regulation [17]. Therefore, the investigation of the SCFA distribution profile will provide deep insight into the microbial metabolism and the gut microbiota regulation potential of black rice. As shown in Figure 3, the yield of total SCFAs, acetic acid, propionic acid, and butyric acid in all samples was obviously increased with the extension of fermentation time. In addition, their contents in the black rice groups were significantly higher than those in the blank control group, indicating the probiotic properties of black rice. After 24 h of fermentation, the total SCFAs in EBR (25.3 mM) and FEBR (24.4 mM) were significantly higher than those in CBR (24.0 mM) and FCBR (23.0 mM) (Figure 3A), suggesting that extruded black rice was more conducive to the production of SCFAs than traditionally cooked black rice. The possible explanation was that the insoluble dietary fiber in black rice was degraded and then transferred into soluble dietary fiber during extrusion. Consequently, the swelling capacity of dietary fiber was increased, and the dietary fiber became more fermentable, promoting the production of SCFAs [27].

As depicted in Figure 3B, no significant difference was observed in the contents of acetic acid among different black rice groups after 24 h of in vitro fecal fermentation. In addition, it has been reported that propionic acid can be rapidly absorbed by intestinal cells and then inhibit cholesterol synthesis by reducing the activities of key enzymes involved in cholesterol synthesis [28]. The results in Figure 3C showed that the contents of propionic acid were 2.83, 2.74, 2.85, and 3.01 mM, respectively, in CBR, FCBR, EBR, and FEBR, in which the contents in FEBR were significantly higher than those in other black rice groups. Moreover, butyric acid is reported to be the energy source of colonic epithelium and beneficial to the prevention of inflammatory responses [29]. It could be seen in Figure 3D that the contents of butyric acid in FCBR (0.76 mM) and FEBR (0.76 mM) were significantly higher than those in CBR (0.49 mM) and EBR (0.55 mM). The above results clearly demonstrated that FEBR showed great advantages over other black rice groups in the production of propionic acid, butyric acid, and total SCFAs during in vitro fecal fermentation, which may exhibit great potential in the modulation of gut microbiota.

#### 3.2.3. Alpha Diversity of Gut Microbiota

**Figure 4:**
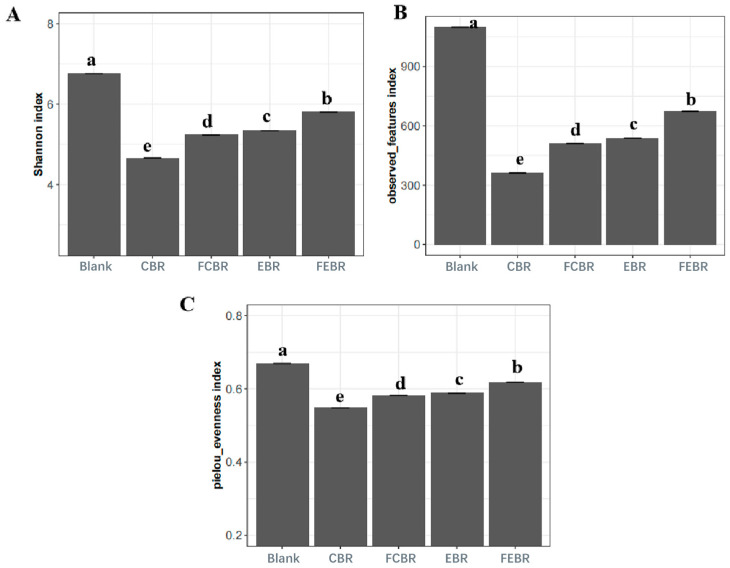
Alpha diversity analysis of gut microbiota after 24 h of in vitro fecal fermentation. (**A**) Shannon index; (**B**) observed features index; (**C**) Pielou’s evenness index. Values labeled with different letters differ significantly (*p* < 0.05).

The alpha diversity, as reflected by the Shannon index (Figure 4A), the observed features index (Figure 4B), and Pielou’s evenness index (Figure 4C), is an important indicator of intestinal homeostasis. Generally, the Shannon index indicates the richness and evenness of the microbiota, and the observed features index reflects the diversity of the microbial community, while Pielou’s evenness index refers to their ratios. The decrease in alpha diversity is generally regarded as characteristic of disease-associated microbiome states [17]. It can be seen in Figure 4 that all the above indices were the highest in the blank control group after 24 h of fermentation, followed by different black rice groups, indicating that the alpha diversity was decreased with the inoculation of black rice samples. It has been reported that alpha diversity decreased significantly after fermentation of highly fermented fibers (e.g., synthetic or isolated fibers) [17]. In addition, the phytochemicals in black rice might also decrease the diversity of the microbial community due to their antibacterial effect [30]. Moreover, black rice samples after solid-state fermentation showed higher alpha diversity than their unfermented ones. Particularly, FEBR showed significantly higher alpha diversity than other black rice groups. It might be because the phenolics in FEBR were dialyzed out the most during in vitro gastrointestinal digestion, and then the chyme of FEBR showed higher fermentability than other black rice groups during in vitro fecal fermentation as aforesaid in Section 3.2.2.

#### 3.2.4. Composition Analysis of Gut Microbiota

**Figure 5:**
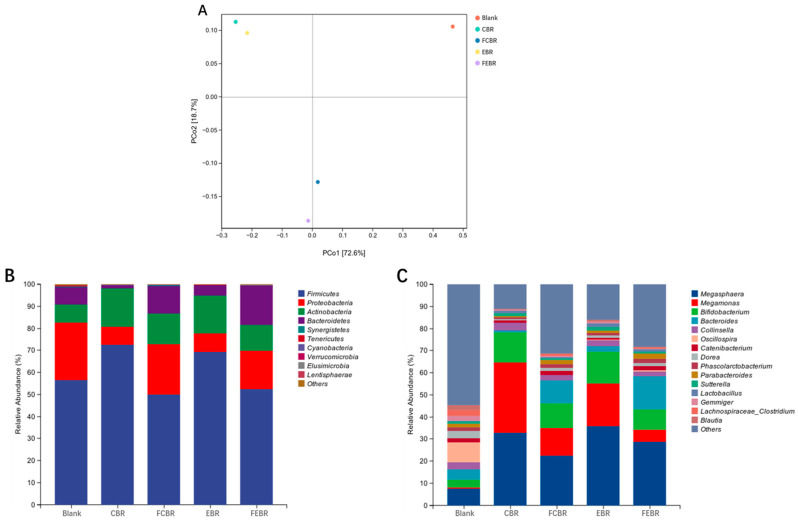
The composition of gut microbiota after 24 h of in vitro fecal fermentation. (**A**) Bray–Curtis PCoA of microbial community; (**B**) relative abundances of microbiota at phylum level; (**C**) relative abundances of microbiota at genus level.

As shown in Figure 5A, the PCoA plot based on Bray–Curtis distances reveals the composition differences in gut microbiota inoculated with different black rice samples after 24 h of in vitro fecal fermentation. The total variance contribution rates of PCo1 (72.6%) and PCo2 (18.7%) were 91.3%, indicating the feasibility of the present analysis. In addition, individual difference was observed between the blank control group and the black rice groups. It was found that both CBR and EBR were positioned at the top left, while FCBR and FEBR were located directly below the figure. The above results clearly demonstrated that the influence of solid-state fermentation pretreatment on gut microbiota composition was significantly greater than that of traditional cooking and extrusion pretreatments during in vitro fecal fermentation of black rice.

The microbiota composition was also compared at the phylum and genus levels to have a better understanding of the gut microbiota modulation potential of different black rice samples, and the results are given in Figure 5B,C. As depicted in Figure 5B, *Firmicutes*, *Proteobacteria*, *Actinobacteria*, and *Bacteroidetes* primarily contributed to the top four phyla in the microbial community of all groups, which was similar to the results of a previous study [15]. Proteobacteria encompass numerous well-known pathogenic strains (e.g., *Salmonella*, *Shigella*, *Helicobacter*, and *Escherichia coli*), which usually induce an imbalance of the intestinal microbiota and chronic colitis [31]. Therefore, the decrease in relative abundances of *Proteobacteria* suggested that black rice inoculation could inhibit the growth of pathogenic microorganisms. This funding was consistent with the results of Alpha diversity as aforesaid, which might be due to the antibacterial effects of the phytochemicals in black rice. *Firmicutes* present the highest proportion among all groups, and it has been reported to be beneficial to immune function and the absorption and synthesis of nutrients [15]. Notably, the relative abundance of *Firmicutes* was increased in the CBR and EBR group but slightly decreased in the FCBR and FEBR group. *Bacteroidetes* encompasses protective and nutritional roles in the development and upkeep of epithelial cell surface homeostasis [15], and its relative abundance was increased in the FCBR and FEBR group but decreased in the CBR and EBR group. Furthermore, the ratio of *Firmicutes*/*Bacteroidetes* (F/B) is reported to be related to metabolic disorders and obesity, and a lower ratio indicates better beneficial effects for obesity prevention [32]. The F/B values were 54.2, 4.0, 15.0, and 3.0, respectively, in CBR, FCBR, EBR, and FEBR, indicating that fermented black rice samples showed greater potential than unfermented ones in obesity prevention through the modulation of gut microbiota, and FEBR was the most valuable.

The relative abundance of gut microbiota at the genus level is presented in Figure 5C, in which *Megasphaera*, *Megamonas*, *Bifidobacterium*, *Bacteroides*, and *Collinsella* contributed the top five genera of all groups. *Bifidobacterium* is one of the well-known probiotics and plays an important role in the enhancement of immunity and the treatment of intestinal diseases (e.g., diarrhea, constipation, and irritable bowel syndrome) [33]. The relative abundance of *Bifidobacterium* in all black rice groups was increased when compared with that of the blank control group, suggesting the probiotic properties of black rice. In addition, *Bacteroides* is regarded as a promising candidate for next-generation probiotics due to its ability to modulate inflammatory responses [27]. It can be seen in Figure 5C that the relative abundance of *Bacteroides* was the highest in FEBR, followed by FCBR, while the CBR and EBR showed a lower relative abundance of *Bacteroides*, indicating the great probiotic potential of fermented black rice, and FEBR was preferred.

#### 3.2.5. Differential Analysis of the Microbial Community

**Figure 6:**
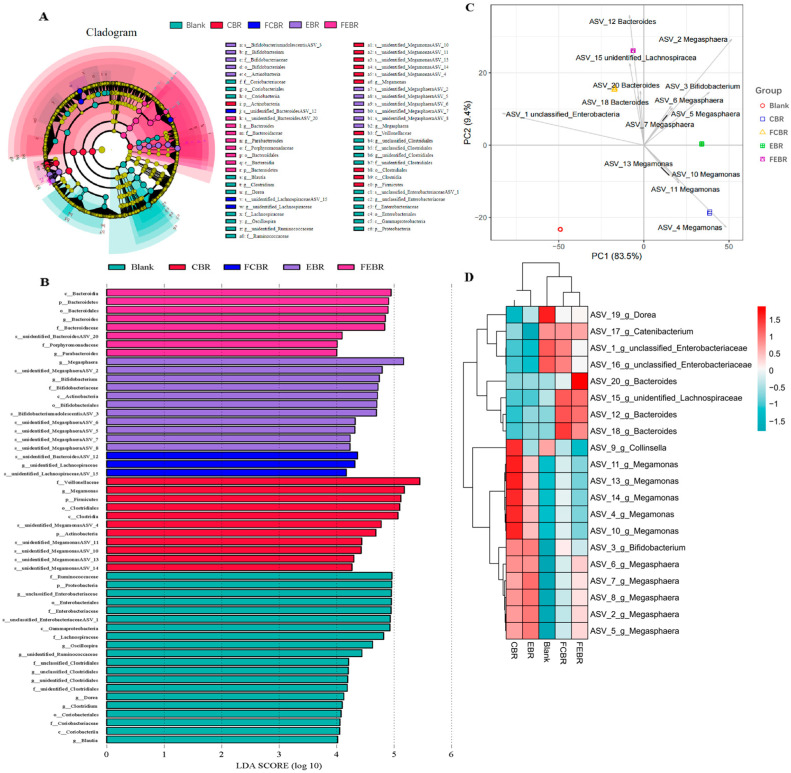
Differential analysis of the key bacteria after 24 h of in vitro fecal fermentation. (**A**) Cladogram of LEfSe analysis; (**B**) plot of LDA scores (LDA score > 4.0); (**C**) PCA analysis; (**D**) clustering heat map analysis.

The LEfSe analysis with a linear discriminant analysis (LDA) score greater than 4.0 was used to discriminate the key bacteria responsible for different black rice groups after 24 h of in vitro fecal fermentation, and the results are shown in Figure 6A,B. It was found that CBR exhibited an enrichment of genus *Megamonas*, and EBR showed an enrichment of genus *Bifidobacterium* and *Megasphaera*. As a comparison, unidentified *Lachnospiraceae* were more abundant in FCBR, while *Bacteroides* and *Parabacteroides* were more abundant in FEBR. Bacteria strains belonging to *Bacteroides*/*Parabacteroides* have been reported to be the major degraders of high-fiber diets and complex polysaccharides (e.g., heteroxylans) [27], while members of the *Lachnospiraceae* family could utilize acetate for butyrate generation [34]. Thus, their increased abundance in FCBR and FEBR may be responsible for the accumulation of butyric acid (Figure 3D).

In addition, the differential bacteria were also analyzed through Principal Component Analysis (PCA) at the Amplicon Sequence Variant (ASV) level, and the results are shown in Figure 6C. PC1 (83.5%) and PC2 (9.4%) explained 92.9% of the variance of the samples. In addition, unfermented black rice groups (CBR and EBR) mainly appeared at the positive direction of the PC1 axis, while fermented black rice groups (FCBR and FEBR) were mostly pointing at the negative direction of the PC1 axis, and all black rice groups differed along the PC2 axis. These results were consistent with those of PCoA, indicating that solid-state fermentation pretreatment induced significant differences in microbial community during in vitro fecal fermentation of black rice. Moreover, it could be found that the abundance of ASV-4 (*Megamonas*) and ASV-3 (*Bifidobacterium*) were distinguished in CBR and EBR, respectively, while the abundance of ASV-15 (unidentified *Lachnospiraceae*) and ASV-12 (*Bacteroides*) were dominated in FCBR and FEBR, respectively.

Afterwards, the top 30 ASVs in terms of abundance ranking after 24 h of in vitro fecal fermentation were extracted, and their differences in abundance were centralized and visualized on a heatmap (Figure 6D). It could be seen that FCBR and FEBR were clustered into the same class, while CBR and EBR were both clustered into another class, which was consistent with the results of PCoA and PCA as aforesaid. Furthermore, it was also found that the proliferation of *Megamonas* in the CBR group was primarily promoted, followed by *Megasphaera* and *Bifidobacterium*. *Megasphaera* proliferation was primarily promoted in the EBR group, followed by *Bifidobacterium* and *Megamonas*. However, the FCBR group primarily promoted the proliferation of *Bacteroides*, followed by *Lachnospiraceae*, *Enterobacteriaceae*, and *Catenibacterium*. The FEBR group primarily promoted the proliferation of *Bacteroides*, followed by *Lachnospiraceae*, *Catenibacterium*, and *Megasphaera* during in vitro fecal fermentation.

## 4. Conclusions

E-SSF showed great potential in the improvement of phenolic bioaccessibility and gut microbiota modulation potential of black rice in present study. The results of in vitro simulated gastrointestinal digestion indicated that EBR and FEBR showed significantly higher bioaccessible phenolics, flavonoids, antioxidant activities, and total phenolic acids than that of CBR and FCBR. Particularly, FEBR showed the highest phenolic bioaccessibility, indicating the great potential of E-SSF in improving phenolic digestibility and bioaccessibility. Furthermore, the intake of the above black rice samples was beneficial to the gas production and SCFA accumulation during in vitro fecal fermentation. FCBR and FEBR inoculation induced significantly lower gas production and higher pH than that of CBR and EBR. In addition, the results of the gas chromatograph confirmed that FEBR showed great advantages over other black rice group in the production of propionic acid, butyric acid, and total SCFAs. Consequently, the results of 16S rRNA amplicon sequencing demonstrated that microbiota composition was significantly influenced by different black rice sample inoculation. Black rice after SSF might increase alpha diversity and *Bacteroidetes* abundance but decrease *Firmicute* abundance during in vitro fecal fermentation, and the FEBR group showed the highest alpha diversity and *Bacteroide* abundance. The above results clearly demonstrated that SSF was beneficial for improving the probiotic potential of black rice, and E-SSF was the preferred. This study will provide a favorable strategy to improve the functional properties of black rice and then promote its consumption and application in the food industry. Future research could involve different black rice genotypes and animal or human experiments to confirm the effects of SSF and E-SSF on the functional properties of black rice.

## Figures and Tables

**Table 1:** The bioaccessible phenolic acids of different black rice samples during in vitro intestinal digestion.

Stage	Phenolic Acid	Samples			
		CBR	FCBR	EBR	FEBR
Intestinal	*p*-hydroxybenzoic acid	662.56 ± 22.64 ^a^	670.82 ± 15.16 ^a^	658.21 ± 16.61 ^a^	664.40 ± 23.01 ^a^
	vanillic acid	115.71 ± 2.76 ^c^	126.77 ± 2.10 ^b^	123.38 ± 2.55 ^b^	144.79 ± 5.54 ^a^
	caffeic acid	75.17 ± 0.38 ^c^	83.37 ± 0.36 ^a^	76.72 ± 0.70 ^b^	71.76 ± 0.52 ^d^
	syringic acid	21.75 ± 0.99 ^b^	20.77 ± 0.09 ^c^	24.06 ± 0.32 ^a^	24.32 ± 0.12 ^a^
	*trans*-*p*-coumaric acid	2.10 ± 0.12 ^c^	2.09 ± 0.12 ^c^	4.95 ± 0.16 ^a^	4.05 ± 0.52 ^b^
	*trans*-ferulic acid	38.96 ± 0.13 ^c^	38.37 ± 0.28 ^c^	66.60 ± 0.63 ^a^	45.45 ± 0.65 ^b^
	*trans*-sinapic acid	39.05 ± 0.34 ^c^	46.23 ± 2.45 ^b^	39.81 ± 0.99 ^c^	49.22 ± 1.19 ^a^
	total	955.31 ± 22.40 ^b^	988.43 ± 15.87 ^ab^	993.72 ± 18.68 ^ab^	1003.99 ± 29.20 ^a^
Dialysate	*p*-hydroxybenzoic acid	27.79 ± 1.73 ^a^	29.12 ± 2.25 ^a^	28.82 ± 1.28 ^a^	30.83 ± 1.28 ^a^
	vanillic acid	5.18 ± 0.27 ^a^	5.35 ± 0.08 ^a^	5.38 ± 0.44 ^a^	5.81 ± 0.66 ^a^
	caffeic acid	5.33 ± 0.12 ^c^	6.43 ± 0.08 ^b^	6.84 ± 0.26 ^a^	6.34 ± 0.25 ^b^
	syringic acid	1.00 ± 0.03 ^a^	0.78 ± 0.03 ^b^	0.75 ± 0.03 ^b^	0.98 ± 0.03 ^a^
	*trans*-*p*-coumaric acid	0.28 ± 0.01 ^a^	0.15 ± 0.01 ^c^	0.20 ± 0.03 ^b^	0.23 ± 0.01 ^b^
	*trans*-ferulic acid	1.85 ± 0.05 ^b^	1.41 ± 0.04 ^c^	1.36 ± 0.04 ^c^	3.01 ± 0.14 ^a^
	*trans*-sinapic acid	1.65 ± 0.01 ^b^	1.54 ± 0.02 ^c^	1.49 ± 0.02 ^c^	2.00 ± 0.05 ^a^
	total	43.07 ± 2.20 ^b^	44.78 ± 2.47 ^b^	44.85 ± 1.93 ^b^	49.20 ± 2.37 ^a^

CBR, cooked black rice; EBR, extruded black rice; FCBR, fermented cooked black rice; FEBR, fermented extruded black rice, the same below; values with different superscripts in the same row differ significantly (*p* < 0.05).

## Data Availability

The raw data supporting the conclusions of this article will be made available by the authors on request.
